# Microevolution of the pathogenic yeasts *Candida albicans* and *Candida glabrata* during antifungal therapy and host infection

**DOI:** 10.15698/mic2019.03.670

**Published:** 2019-02-08

**Authors:** Pedro Pais, Mónica Galocha, Romeu Viana, Mafalda Cavalheiro, Diana Pereira, Miguel Cacho Teixeira

**Affiliations:** 1 Department of Bioengineering, Instituto Superior Técnico, Universidade de Lisboa, Lisboa, Portugal.; 2 iBB - Institute for Bioengineering and Biosciences, Biological Sciences Research Group, Instituto Superior Técnico, Lisboa, Portugal.

**Keywords:** fungal pathogens, hostpathogen interaction, microevolution, virulence, biofilm formation, antifungal resistance

## Abstract

Infections by the pathogenic yeasts *Candida albicans* and *Candida glabrata* are among the most common fungal diseases. The success of these species as human pathogens is contingent on their ability to resist antifungal therapy and thrive within the human host. *C. glabrata* is especially resilient to azole antifungal treatment, while *C. albicans* is best known for its wide array of virulence features. The core mechanisms that underlie antifungal resistance and virulence in these pathogens has been continuously addressed, but the investigation on how such mechanisms evolve according to each environment is scarcer. This review aims to explore current knowledge on micro-evolution experiments to several treatment and host-associated conditions in *C. albicans* and *C. glabrata*. The analysis of adaptation strategies that evolve over time will allow to better understand the mechanisms by which *Candida* species are able to achieve stable phenotypes in real-life scenarios, which are the ones that should constitute the most interesting drug targets.

## INTRODUCTION

Infections by fungal pathogens have become a relevant health problem, especially for the increasing immunocompromised population [1]. Infections by *Candida* species are the most common cause of fungal infections and represent the 4^th^ leading cause of hospital acquired bloodstream infections in the USA [2–4]. *Candida albicans* and *Candida glabrata* represent the two most commonly isolated species worldwide [2, 5].

Despite representing the bulk of *Candida* infections, each species possesses quite different traits in terms of antifungal susceptibility profiles and virulence features. *C. glabrata* presents high levels of intrinsic and acquired resistance to azole antifungals, especially due to overexpression of multidrug resistance transporters activated by the transcription factor Pdr1 [6–9]; while *C. albicans* isolates are usually more susceptible to azole treatment [10]. On the other hand, *C. albicans* carries a number of virulence features that are absent in *C. glabrata*, such as the formation of hyphae. Hyphal formation plays an important part in colonization and biofilm formation, which is consistent with the notion that *C. albicans* biofilms are bulkier than the ones formed by *C. glabrata* [11]. Moreover, hyphae contribute for tissue invasion and phagocyte escape [12–15]. *C. glabrata* mechanisms of tissue invasion are mostly unknown; although it is hypothesized to possibly occur by endocytosis induction of host cells [16]. As for phagocyte escape, *C. glabrata* applies a persistence strategy by replicating inside phagocytes and eventually leading to cell lysis due to fungal load [17, 18], rather than actively escaping. The production of secreted aspartyl proteases (SAPs) is another critical virulence trait in *C. albicans*, allowing the pathogen to degrade host barriers and invade surrounding tissue [19]. In turn, *C. glabrata* does not appear to produce significant levels of proteinase activity [20] nor to induce significant tissue damage [16]. However, *C. glabrata* possesses a family of aspartic proteases, which is mainly associated with cell wall remodeling and possible immune evasion [21]. In addition, the expression of phospholipases is yet another feature that allows *C. albicans* to acquired nutrients in host nutrient-poor niches and contributes to invasion, whereas *C. glabrata* shows a very low level of phospholipase activity [20].

This review aims to explore the data retrieved from microevolution experiments performed on both *C. albicans* and *C. glabrata*. Comprehensive knowledge about the mechanisms of antifungal resistance and virulence, their clinical prevalence and strategies to counteract them has been duly explored. However, the way such mechanisms evolve during colonization of the human host and upon confrontation with antifungal treatment can provide valuable insight on the pathogenic nature of *Candida* spp. employed in the clinical setting. By better understanding the way *Candida* spp. evolve in distinct environments and selective pressures, it could be possible to delineate better strategies to tackle infections by these pathogens.

## *CANDIDA* EVOLUTION TOWARDS DRUG RESISTANCE

### Antifungal drugs and resistance mechanisms in *C. albicans* and *C. glabrata*

Azoles are the common first-line drugs against most *Candida* species because of their safety profile and availability in both oral and intravenous formulations [22]. They act by inhibiting the 14α-demethylase Erg11 in the ergosterol biosynthesis pathway and cause the accumulation of the toxic sterol 14,24-dimethylcholesta-8,24(28)-dien-3β,6α-diol (DMCDD) that permeabilizes the plasma membrane [23]. Nevertheless, the fungistatic nature of azoles imposes strong directional selection for the evolution of resistance. Additionally, some *Candida* species, such as *C. glabrata*, are intrinsically less susceptible to this class of antifungal drugs. In fact, *C. glabrata* has risen dramatically in frequency as a significant cause of blood stream infection (BSI) since the introduction of azole drugs in the 1980s [24]. The increase in the prophylactic use of azoles for high-risk individuals undoubtedly contributed to the increasing development of *C. glabrata* resistance to these antifungal drugs, which are significantly effective in eradicating infections caused by other *Candida* species [25–27]. Still, these anti-fungals are inactive against biofilm-associated infections, which is a significant public health problem due to the increasing usage of medical devices [28].

*C. albicans* might develop resistance toward azoles through upregulation of efflux pumps Cdr1, Cdr2 and Mdr1, inactivation of Erg3 that synthesizes the toxic sterol DMCDD, and upregulation or mutations in the gene encoding azoles target, *ERG11* [29, 30]. Generally, the upregulation of drug efflux pumps and drug target is the result from point mutations in genes encoding the regulators of their expression [31–36], or from increased copy number of the genes through genome rearrangements such as whole chromosome and segmental aneuploidies [37–39]. Moreover, it was very recently demonstrated that *C. albicans* can also gain azole resistance by altering sphingolipid composition, *in vitro* [40].

In contrast to what is observed in *C. albicans* and despite the potential for *ERG11* point mutations to have a greater impact in haploid organisms, as is the case of *C. glabrata*, several studies suggest that mutations in *ERG11* are not involved in clinical azole resistance in this pathogen [8, 41, 42]. The major described mechanism of acquired azole resistance in *C. glabrata* clinical isolates is the increased drug efflux due to the upregulation of drug efflux pumps [43–46]. This is generally caused by gain-of-function (GOF) mutations within the gene encoding the key transcriptional regulator of drug resistance, *PDR1*, responsible for the upregulation of the drug efflux pumps, Cdr1, Cdr2, Snq2 and Qdr2, which directly confer most of the acquired azole resistance in this pathogen and, surprisingly, was also found to enhance virulence [41, 46–48]. A high frequency of acquired azole resistance *in vitro* in *C. glabrata* populations has been linked to a loss of mitochondrial function, which leads to the upregulation of ABC transporter genes [47, 49]. In fact, this phenotype is associated with Pdr1 expression, as mitochondrial dysfunction was shown to increase the expression of *PDR1*, further exacerbated by a positive auto-regulatory loop that leads to *PDR1* and target genes overexpression [6, 50]. It was proposed that this pathogen can switch between states of mitochondrial competence (azole-susceptible) and incompetence (azole-resistant) in response to azole exposure, probably through chromatin epigenetic modifications [51]. Until recently, clinical relevance of mitochondrial mutants was questionable in light of their decreased fitness. Nevertheless, Ferrari *et al*. found that an azole-resistant *C. glabrata* clinical isolate not only exhibited mitochondrial dysfunction and upregulation of *CDR1* and *CDR2*, but also was more virulent than its susceptible counterpart in both systemic and vaginal murine infection models [52]. This report demonstrated that mitochondrial dysfunction can confer selective advantage under host conditions. Calcium signaling was also proposed to play a role in azole resistance, since the loss of Ca^2+^ signaling pathway changes fluconazole activity from fungistatic to fungicidal *in vitro* [51]. Furthermore, in very recently published data, at least 78 other genes were suggested to be involved in *C. glabrata* resistance toward fluconazole and voriconazole [53]. This points to the hypothesis that there might be other yet unknown alternative paths to azole acquired resistance in this pathogen.

Echinocandins are the only new class of antifungals to reach the clinic in decades, with three echinocandins currently available for clinical use: caspofungin, micafungin, and anidulafungin [26, 30]. These antifungals target the fungal cell-wall by acting as noncompetitive inhibitors of β-(1,3)-D-glucan synthase enzyme complex which catalyzes the production of glucan, the major component in *Candida* cell walls [26, 54]. The disruption of (1,3)-β-D-glucans impairs the structure of growing cell walls, resulting in loss of structural integrity, osmotic instability and cell death. Therefore, a fungicidal effect is accomplished by disrupting cell wall synthesis. More important, not only echinocandins present good safety profiles and their toxicity is very low due to their unique target, that is absent in mammalian cells, but also have been shown to have activity against *Candida* biofilms as the inhibition of polysaccharide production could lead to lysis and dissolution of the extracellular matrix (ECM) [55, 56]. This is an extremely important feature since biofilm-associated infections are very hard to treat and are recurrent in patients with medical devices such as pacemakers or catheters. Considering all the advantages, the Infectious Diseases Society of America guidelines currently favor echinocandins as first-line treatment for systemic candidiasis in patients with moderate-to-severe infection and in those with prior exposure to azoles, with fluconazole held in reserve for the treatment of patients with less severe infections [57]. Moreover, the European Society of Clinical Microbiology and Infectious Diseases recommends echinocandins as first-line treatment for all patients with systemic candidiasis [58]. Even so, *Candida* clinical isolates exhibiting reduced echinocandin susceptibility have been found over the past years [59–61]. In *C. albicans*, this reduced susceptibility is strictly attributed to changes in the subunit of glucan synthase enzyme complex Fks1, which is the specific target of echinocandins. Indeed, genetically related isolates from the same patient with different mutations in *FKS1* were identified, suggesting that reduced susceptibility can evolve in the patient [60, 62]. Otherwise, mutations in both Fks1 and its paralog Fks2 (but not Fks3) have been associated with resistance in *C. glabrata* [63]. Interestingly, recent studies reported that echinocandin resistance is more common in *C. glabrata* compared with other species and that this rate can be attributed to the high potential of *C. glabrata* for developing resistance mutations [64, 65]. In 2011, Costa-de-Oliveira *et al*. reported for the first time the *in vivo* acquisition of echinocandin resistance following anidulafungin therapy in a patient with *C. glabrata* invasive candidiasis, highlighting the need for antifungal susceptibility surveillance in patients under extended echinocandin therapy [61].

Polyenes, such as amphotericin B (AmB), are fungicidal drugs that act by binding to ergosterol in the lipid layer. The typical mode of action associated with polyenes is the resulting formation of pores in the cell membrane, ultimately leading to fungal cell permeabilization [23]. Additionally, merely the binding of AmB to ergosterol was shown to kill yeast cells, with pore formation being a complementary mechanism [66]. These antifungals have the broadest spectrum of activity compared to any other anti-fungal molecules. However, polyenes are highly toxic since they have a lower but non-negligible affinity for cholesterol, the mammalian membrane sterol, which is responsible for numerous side effects. These side effects of therapy with polyenes, namely AmB, are substantial and may be divided into acute (fever, vomiting, headache) and sub-acute (kidney problems or failure) [67]. Despite the noteworthy disadvantages, AmB is still used for the treatment of the most serious fungal infections, in large part due to its broad spectrum of activity. However, resistance toward this anti-fungal has been found in many clinical isolates, including *C. glabrata* [68–70]. The molecular mechanisms underlying polyene resistance are poorly documented, especially in pathogenic yeasts. It is thought that a decrease in the levels of ergosterol in the cell membrane is connected to the resistant phenotype [70, 71].

Pyrimidine analogs such as flucytosine were first used to treat fungal infections in the 1960s. These antifungals have fungistatic activity exerted through the interference with pyrimidine metabolism, as well as RNA/DNA and protein synthesis. Yet, the quick inception of resistance banned the use of flucytosine as a monotherapy, and consequently it is only used in combination with other anti-fungals [72]. Decreased susceptibility to the most used pyrimidine, flucytosine, has been related with point mutations in *FUR1, FCY1* (encoding enzymes involved in the pyrimidine pathway) and *FCY2* (encoding a cytosine permease) genes in both *C. albicans* and *C. glabrata* [73, 74]. Moreover, it was recently discovered in our lab that the deletion of *FPS1* or *FPS2*, encoding aquaglyceroporins, leads to an increased accumulation of flucytosine within *C. glabrata* cells [75].

Considering the intrinsic variation regarding drug susceptibility among different *Candida* species and the increasing acquired resistance in several clinical isolates, it becomes evident that the genetic evolution underlying drug resistance phenomenon must continue to be investigated. Unveiling new potential targets is imperative to develop new suitable strategies to fight increasing *Candida*-associated infections and, although the nature of intrinsic resistance is unknown, the development of resistance can be studied.

### Evolution of *C. albicans* and *C. glabrata* resistance toward antifungal drugs

The acquisition of resistance depends on various factors and differs severely between species, cell populations and the imposed stress. Genome dynamics and evolution according to the cell's needs is the process through which cells are able to adapt and survive in hostile environments, such as the presence of drugs. Evolutionary changes in organismal traits may occur either gradually or suddenly and encompasses single-point mutations; gene duplications, deletions, inversions, and insertions; chromosomal rearrangements; aneuploidies; the loss of heterozygosity (LOH, in diploid organisms); and horizontal gene transfer and/or hybridization [76]. Noteworthy, recent advances in DNA sequencing technologies have now made it possible to identify genetic changes between ancestral and derived organisms on a whole-genome scale for any species. Therefore, adaptative evolution experiments can provide insights into both the genetic basis and dynamics of adaptation to a specific environment.

**Figure 1:**
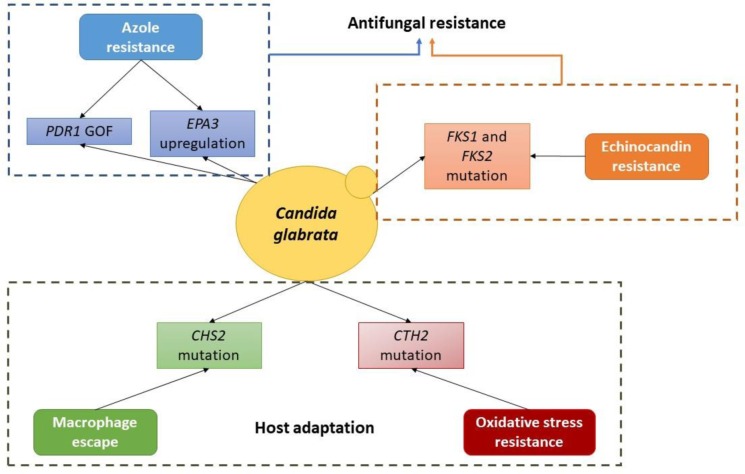
Schematic depiction of the currently known molecular basis of evolution towards antifungal resistance and host adaptation in *C. glabrata*.

The emergence of adaptative mutations is unpredictable and poses genetic variability within the cell population. Genetic adaptation is especially rapid when microbial populations are introduced into new environments [77]. For instance, antifungal treatment exerts selective pressure to which only the cells that acquire resistance-related mutations will be able to survive. However, fungistatic drugs favor the occurrence of resistance-related mutations rather than fungicidal ones. The fact that these drugs do not lead to cell death, instead their action results in growth arrest, allied to the high genome plasticity of *Candida* species enables cells to adapt to the new hostile environment through genetic modifications. Afterwards, selective pressure will select those that acquired resistance mutations to survive and give rise to resistant progeny therefore propagating the resistant phenotype in the population. Likewise, the drug-dosage regime is an important feature that influences the rate of resistance achievement. It was seen *in vivo* that less frequent administration of high drug dosages leads to faster emergence of resistance rather than the application of more periodic lower dosages [78]. Nonetheless, regardless of the rate at which resistance to the different antifungal classes arises, resistance to all classes of antifungals has been reported in both the laboratory and clinical settings [26]. Occasionally, within a clonal cell population, there is significant variability in the response to anti-fungals. This is called heteroresistance and constitutes a very important trait of pathogenic fungal populations as heteroresistant strains are not detected in standard susceptibility assays and may be a driver of azole resistance and therapy failure [79].

**Figure 2:**
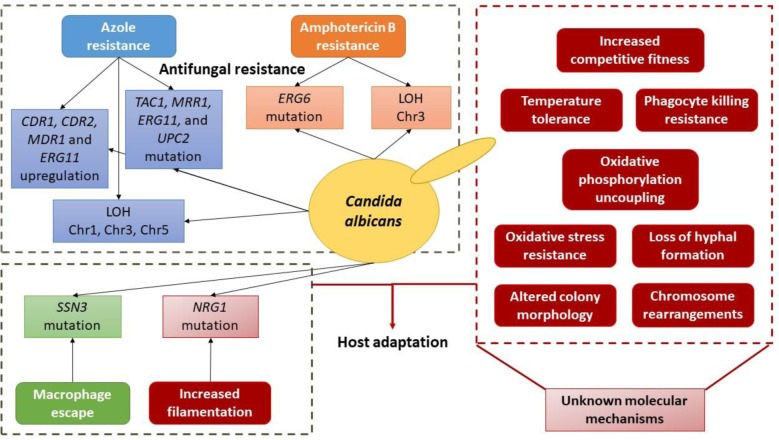
Schematic depiction of the currently known molecular basis of evolution towards antifungal resistance and host adaptation in *C. albicans*.

*In vivo* studies of sequential clinical isolates to monitor the evolution of antifungal resistance complemented by *in vitro* experimental evolution is a powerful approach to unveil the mechanisms underlaying the development of antifungal resistance in different species against specific antifungals in a timescale. This allied with next-generation sequencing (NGS) and comparative genomics not only enable the detection of footprints of genetic evolution, but also are powerful tools to unveil the genomic changes accompanying the emergence of resistance. Some studies probing azole and echinocandin resistance mechanisms in the clinical setting have shown the existence of resistant isolates that do not display typical resistance hallmarks (e.g., *PDR1* GOF or *FKS* mutations) [80–82]. Microevolution studies can contribute to better elucidate the evolution of additional resistance mechanisms that can occur in the clinic environment. Furthermore, these studies can provide a way to investigate population dynamics and hetero-resistance, what would add value to current knowledge on how initial and transient resistance mechanisms arise.

### Evidences of resistance evolution: *in vitro*

*In vitro* evolution experiments provide a framework for understanding genome-wide changes that occurred during cell adaptation. Although the clinical relevance of *in vitro* studies is questionable in light of the feeble mimicking of human host environment, they have several associated advantages: controlled experimental conditions make the processes reproducible and facilitate the identification of factors that drive drug resistance; it is possible to have real-time control of new emergent features; easy replication allows the sample size to be greater and so genetic drift is minimized, since the population size at each transfer must be large enough to avoid beneficial mutations that arise to be lost to random drift [77]. These *in vitro* evolution experiments coupled with whole genome sequence techniques constitutes a powerful tool to address acquired resistance phenomena as it is possible to have a genome-wide view of adaptation and drug resistance progress and establishment.

*In vitro* evolution experiments might be carried out following a serial dilution system or a continuous system. The serial dilution system is the most used method due not only to its lower cost, but also to the feasibility in conducting an experiment that requires such a high number of replicates in parallel. Routinely, cells are transferred by serial dilution of a stationary-phase culture into fresh medium containing a concentration of drug that is inhibitory but not lethal [83]. For instance, Cowen *et al*. monitored the development of resistance toward fluconazole in replicated experimental populations founded from a single, drug-sensitive *C. albicans* cell for 330 generations [83]. Their results demonstrated that not only all populations exposed to fluconazole were able to adapt to its presence, but also that the development of drug resistance followed different trajectories in the initially identical populations. The expression profile of genes known to be involved in azole resistance (*CDR1, CDR2, MDR1* and *ERG11*) was different within the resistant populations, as well as the level of resistance. Moreover, other genomic changes such as alterations in DNA fingerprints were detected in the resistant populations, although their connection with drug resistance is not known. Overall, the authors concluded that chance, in the form of mutations that confer an adaptive advantage, is a determinant in the evolution of drug resistance in experimental populations of *C. albicans*.

*C. albicans* resistance development toward the fungicidal AmB has also been studied *in vitro*. Vincent *et al*. sequenced sequentially laboratory evolved strains and observed that mutations in *ERG6* followed by loss of heterozygosity of chromosome 3, where *ERG6* resides, were behind AmB resistance acquisition [84]. Nevertheless, this acquired resistance came at a great cost. AmB-resistant mutants were hypersensitive to oxidative stress, febrile temperatures, and killing by neutrophils and had defects in filamentation and tissue invasion. Furthermore, the authors saw that these strains were avirulent in a mouse infection model. Despite the fact that costs of evolving resistance to AmB limit the emergence of this phenotype in the clinic, resistant clinical isolates of both *C. albicans* and *C. glabrata* have been found over the past years [68–70].

Another example of *in vitro* evolution experiment following a serial dilution system is a very recent study performed in our lab in which an *in vitro* evolution of a *C. glabrata* isolate toward multiazole resistance was carried out. Cavalheiro *et al*. analyzed the changes in the genetic profile of a *C. glabrata* isolate from azole susceptibility to posaconazole, clotrimazole, fluconazole and voriconazole resistance, evolved in a stepwise manner induced by longstanding incubation with fluconazole [85]. Remarkably, despite all the evolved populations presented reduced azole accumulation when compared to the parental one, only that displaying resistance toward all azoles acquired a GOF mutation in *PDR1* and therefore upregulation of multidrug resistance transporter genes. Even more interesting was the correlation of drug resistance with increased expression of adhesin genes, namely *EPA3*. The authors demonstrated that the intermediate population displaying resistance toward posaconazole and clotrimazole had increased expression of adhesins and, consequently, an increased ability to adhere to other *C. glabrata* cells and to epithelial cells. Specifically, they showed that the absence of Epa3 adhesin increases *C. glabrata* cells susceptibility to all azoles tested. Epa3 and possibly other adhesins, were proposed to play a role in azole resistance probably by promoting cellular aggregation, which protects the cells from extracellular drug concentration. Interestingly, the overexpression of adhesin-like genes in *C. glabrata* resistant isolates was demonstrated *in vivo* by others [86, 87], but their role in drug resistance was never analyzed until this study that enhances the presence of yet unknown mechanisms driving acquisition of resistance. It was suggested that azole-exposed *C. glabrata* population is iteratively selected toward resistance at minimum cost. Full resistance seems to be acquired through the emergence of *PDR1* GOF mutations and, consequently, overexpression of drug efflux pumps. However, before this, the population transcriptome is remodeled reflecting the transient selection of more-fit subpopulations. When *PDR1* GOF mutations emerge in part of the population, natural selection acts favoring these more-fit cells and ultimately leading to the disappearance of other subpopulations.

Very recently, Bordallo-Cardona *et al*. investigated the ability of five echinocandin-susceptible *C. glabrata* isolates to acquire *in vitro* resistance to anidulafungin and micafungin and, remarkably, all isolates acquired resistance after 2–4 days of exposure to low and constant micafungin concentrations and mutations in *FKS2* were found in all of them [88]. This study highlights the idea that the constant exposure to low doses of echinocandins promotes the development of resistance. Moreover, these results showed the ease of resistance acquirement, even to fungicidal drugs, stressing the need for the development of new anti-fungals to eradicate *Candida* infections, or the design of drugs that target resistance mechanisms.

In contrast with the most used serial dilution system, the continuous culture system implies that the environment and the cell physiological state are maintained constant throughout the course of the experiment. Huang *et al*. reported the first study to investigate the emergence of adaptative traits in *C. albicans* during *in vitro* evolution in continuous cultures under increasing concentrations of fluconazole [89]. They used a visualizing evolution in realtime (VERT) approach that not only enables the monitoring of adaptative events, but also facilitates the identification and isolation of adaptative mutants from the population [90]. Some remarkable findings were that several resistant mutant cells appeared rapidly in independent lineages and that a higher frequency of adaptative events occurred in the presence of the drug. Moreover, data shown in this study sustain the idea that rapid resistance can arise from mutations in subpopulations and also suggest that drug resistance mechanisms are not always related with fitness cost.

### Evidences of resistance evolution: *in vivo*

The most reliable approach to study the evolutionary emergence of drug resistance involves the analysis of isolates from an infected individual over treatment time. However, although this approach is the most clinically relevant, it has some significant constrains such as the small sample sizes. Due to the limited number of clones isolated, the population dynamics during the emergence of drug resistance *in vivo* has been difficult to assess [89]. Also, population parameters such as the genotype of the initial population or the exact number of generations are hard to determine.

Despite these constrains, many efforts have been made to understand the emergence of resistance in fungal pathogens *in vivo*, namely in *Candida* species. More than two decades ago, White *et al*. analyzed a set of 17 isolates of *C. albicans* collected over two years from an HIV-infected patient who was receiving azole treatment for recurrent oropharyngeal candidiasis [91]. The initial isolate in the series was susceptible to fluconazole treatment, but by the end of the two-year period, the strain had acquired a level of resistance 200-fold higher than that of the original isolate. Over the time of sampling, mutations were acquired in the gene encoding the fluconazole target, *ERG11* [92, 93]. Noteworthy, the overexpression of the *CDR1* and *MDR1* efflux pumps was also detected, although the causes were not evaluated in this study [91]. Ten years later, another study addressing this series of isolates (among others) identified GOF mutations in the transcription factor *TAC1* [94], one of which was identified more recently in another isolate as well [95]. The same study also identified GOF mutations in *MRR1* and LOH of the mutated allele was associated with the observed Mdr1 overexpression in these isolates [95]. Yet another study, by Cowen *et al*., used this same set of *C. albicans* isolates to evaluate the impact of the essential chaperone Hsp90 in the natural evolutionary process [96]. Interestingly, they found that inhibition of Hsp90 or of calcineurin decreased fluconazole resistance, more in early isolates rather than in later ones. Drugs structurally related to GdA (Hsp90 inhibitor) are currently undergoing clinical evaluation [97] and present a good alternative to prevent *de novo* evolution of resistance. Hsp90 inhibitors are effective in overcoming fungal drug resistance at concentrations that are clinically well tolerated. In 2011, Hoot *et al*. analyzed the sequence of the transcriptional regulator *UPC2*, that regulates the expression of *ERG11*, in the same 17 isolates from the set and searched for mutations [31]. They found a mutation in *UPC2* that caused the overexpression of *ERG11* contributing for the resistant phenotype [31]. Previous studies probing azole resistance in matched fluconazole-resistant and susceptible *C. albicans* isolates had also described the occurrence of *UPC2* GOF mutations that result in the overexpression of *ERG11* and consequent azole resistance [31, 32].

In 2006, Coste *et al*. matched pairs of azole-susceptible and azole-resistant *C. albicans* clinical isolates were screened for mechanisms that could explain azole resistance [35]. The authors found that GOF mutations in the azole resistance regulator *TAC1*, followed by LOH, was responsible for azole resistance [35]. In 2007, Coste *et al*. analyzed the genetic and genomic events that lead to azole resistance through alteration of *TAC1* and *ERG11* in sequential clinical isolates [94]. Consistent with previous reports addressing the acquisition of azole resistance [91, 98], the development of azole resistance in *C. albicans* was proposed to occur in a stepwise manner, in which the acquisition of mutations in drug resistance genes, such as *TAC1* and *ERG11*, is a prerequisite for the development of azole resistance and is followed by different LOH events, including chromosome loss [94]. These chromosome alterations can be accompanied by acquisition of extra chromosomal elements (isochromosome formation), all contributing to drug resistance in *C. albicans*. The authors proposed that not only the presence of specific mutations in azole resistance genes is necessary but also their increase in copy number by LOH and the addition of extra Chr5 identical copies. Likewise, it was reported that mutations in the multidrug resistance regulator *MRR1* and LOH are the main cause of the multidrug transporter Mdr1 overexpression in fluconazole resistant *C. albicans* strains [34, 99].

In 2015, Ford *et al*. studied the genetic base of yeast adaptation to drug treatment in the human host using whole-genome sequencing of sequential isolates from the same patient [100]. 43 *C. albicans* isolates were taken over time from eleven oral candidiasis patients who were being treated with fluconazole and their genome was sequenced demonstrating frequent LOH and single nucleotide polymorphisms (SNPs) in 166 genes as the main modifications associated with decreased fluconazole susceptibilities. Both chromosome 3, which comprises *CDR1* and *CDR2* (efflux pumps coding genes) and *MRR1* (*MDR1* efflux pump regulator), and chromosome 5, which contains *ERG11* and *TAC1* (positive regulator of *CDR1* and *CDR2*), were found to suffer LOH. Even more interesting, the authors found that mutations in cell adhesion, filamentous growth and biofilm formation related genes occurred in several isolates, which suggests a co-evolution between virulence and resistance. Moreover, a lot of genetic variation was found between samples from the same patient, demonstrating the selective pressure acting on the population.

Recently, Vale-Silva *et al*. analyzed the genome of two *C. glabrata* clinical isolates taken from the same patient, one before (azole susceptible) and another after (azoleresistant) a time-lapse of 50 days of azole therapy [86]. Only 17 nonsynonymous SNPs were found comparing the genomes of both strains, among which a known *PDR1* GOF mutation, and small size indels in coding regions mostly in adhesin-like genes. Interestingly, both isolates carried a nonsynonymous mutation in *MSH2* known to favour the hyper-mutator phenotype (V239L) and the number of accumulated mutations between both clinical isolates was shown to be consistent with the presence of a *MSH2* defect [101]. Nonetheless, the remaining genetic alterations were attributed to fitness or accidental mutations and not to the resistant phenotype, which was exclusively attributed to the *PDR1* GOF mutation. Interestingly, *C. glabrata* virulence and adherence to host cells was also linked to the emergence of *PDR1* GOF mutations in clinical isolates. *PDR1* GOF mutations have been associated with increased *C. glabrata* virulence [48], increased expression of adhesins and increased adherence to host epithelial cells [87, 102].

In 2012, Singh-Babak *et al*. reported the first genomewide analysis of mutations occurring during evolution of echinocandin resistance in a series of *C. glabrata* isolates recovered over a 10-month period from a patient that underwent caspofungin treatment for bloodstream candidemia [103]. Although a nonsynonymous mutation in the drug target gene *FKS2* is enough for the resistant phenotype, an elevated fitness cost was associated with it. Remarkably, further acquired mutations demonstrated to be able to mitigate the fitness cost caused by the *FKS2* mutation. The authors also implicate the molecular chaperone Hsp90 and calcineurin in the resistant phenotype acquired by the *FKS2* mutation [103], however, the inhibition of Hsp90 in *C. glabrata*, as well as in *C. albicans*, decreases basal tolerance to drugs [96, 103–105], and so, a particular role of this pathway in the evolution and acquisition of antifungal resistance should be address with caution.

Otherwise, Biswas *et al*. performed a genome-wide analysis of *C. glabrata* antifungal resistance markers to simultaneously unveil mutation patterns of genes known to be involved in resistance toward different drug classes [106]. NGS was used to unravel SNPs between resistant strains and susceptible counterparts. Although high-quality nonsynonymous SNPs were found in *FKS1* and *FKS2* (echinocandin resistance), *FCY2* (flucytosine resistance), *PDR1* (azoles resistance), the later were found in both azolesusceptible and resistant strains, highlighting the need for further investigation.

## *CANDIDA* EVOLUTION TOWARDS INCREASED BIOFILM FORMATION

Biofilms are organized communities of cells developed after adhesion to a surface and enclosed in an ECM [107, 108]. The ability to grow as biofilms presents an advantage for fungal pathogens, regarding colonization and persistence in different host tissues [109], but also in the resilience against antifungal treatments [110, 111]. This is especially true for *C. albicans* and *C. glabrata*. Both species are known to use biofilms to colonize the surface of several medical devices based on different materials [112]. As a result, biofilm formation comes with strong impact in medicine as well as in the development of improved therapeutic solutions.

Biofilm formation is a multifaceted process well described for *C. albicans* in several *in vitro* studies [109, 112]. It develops through four distinct steps: the early phase, characterized by the adhesion of yeast cells to a surface; the intermediate phase, consisting in cell proliferation and differentiation into pseudohyphae and hyphae, accompanied by the production and secretion of extracellular polymeric substances (EPS); the maturation phase, in which fully-formed three-dimensional biofilm structure surrounded by a thick ECM is reached; and the dispersal phase, where detachment of round budding yeast cells from the biofilm may lead to the formation of biofilms in other host niches [112–114]. Although very simply described herein, the formation of *Candida* biofilms is a very complex process which relies on different mechanisms, such as cell-to-cell adhesion, crucial for the integrity of the biofilm [113], or the formation of the ECM, composed by different EPS, some identified as important for the resistance to antifungal drugs [115].

To tackle *C. albicans* and *C. glabrata* biofilms from a therapeutic point of view, it is necessary to identify which of these mechanisms, responsible for the formation and maintenance of the biofilm, are used *in vivo* to persist in the human host or on medical devices, and if they change overtime during the infection of a given patient, increasing the strain's ability to form bigger, thicker or more stable biofilms. Although few information has been collected regarding these topics, some studies have been focused on biofilm forming ability of *Candida* sequential isolates.

Jain *et al*. assessed the biofilm forming ability of ten sequential urine isolates available from nine patients, taken in a time-range of 70 days [116]. The isolates collected from four of the nine patients were composed of both *C. albicans* and *C. glabrata* species. The single strain isolates revealed no significant variability in biofilm forming ability, even comparing isolates taken with 70 days in between. However, it varied in infections caused by more than one strain [116]. In line with these results, another study reported biofilm forming ability as a specific trait of a given *Candida* isolate that remains stable in serial isolates [117]. Moreover, Bitar *et al*., from 85 clinical isolates obtained from different patients, have identified by multilocus sequence typing (MLST) two isolates that shared homology features, suggesting a possible case of microevolution of one strain that may have spread into another patient. Once again, no variability in the capacity to form biofilms was observed between the two isolates [118]. Although it seems that biofilm formation ability of an isolate does not change often throughout the infection time in a given patient, there are some cases of registered evolution [116], but there is no information on the underlying differences in genome sequence. More studies should be performed to assess these changes that may underly important features of host adaptation. It is important to note that although a given strain does not appear to change its ability to form biofilm over the course of an infection, biofilm formation among distinct isolates is quite disparate. The mechanisms underlying distinct biofilm formation ability across isolates is largely unknown and warrants further investigation.

In addition, it would be interesting to try to evolve the capacity of *Candida* strains to form more biofilm. *In vitro* evolution of *Candida* strains towards higher biofilm formation would help identify the main factors influencing biofilm formation, as well as the more propitious environment (materials and *in vivo* models) for *Candida* biofilms. The study of *Candida* strains in various time-points of biofilm formation and maintenance would enhance our knowledge about the more essential mechanisms for *Candida* biofilms and their persistence and evolution in the human host.

## *CANDIDA* EVOLUTION TOWARDS ADAPTION TO HOST NICHES

Microevolution plays an important role on the remarkable capacity of adaptation of *Candida* species to the many different host niches. Evolution experiments have emerged as a powerful tool for testing specific theoretical models and are a great tool for understanding microevolution and to study factors which are important for local adaptation and for the survival of the pathogens in the host [119, 120].

There are already some studies on microevolution of *C. albicans* in serial passage experiments through different host niches, namely kidney [121] and spleen [121, 122], two main target organs in murine *Candida* sepsis [123]. In Lüttich *et al*., serial passage experiments of the commonly used *C. albicans* strain SC5314 through the murine kidney, showed that despite leading to increased phenotypic variability within the population possibly by microevolution, the overall virulence, fungal fitness and the host response did not follow a clear trend between infected animals, revealing that the *C. albicans* strain used was already well adapted to the murine kidney [124].

However, in another case, serial passage experiments with the same *C. albicans* strain through murine spleens were performed and at the fifth passage a stable respiratory mutant isolate was recovered, showing delayed filamentation initiation and abnormalities in carbon-assimilation. This recovered strain was more resistant to phagocytosis by neutrophils and macrophages and showed attenuated virulence in mice. It was hypothesized that this mutant seems to persist and proliferate without killing the mice. The regulation of respiration was proposed to have influence in the interaction between *C. albicans* and the host, as the mutant displayed uncoupled oxidative phosphorylation, a possible mechanism of adaptation to stressful host environments by *C. albicans* [121].

In Forche *et al*., genome wide genetic and phenotypic evolution were evaluated in *C. albicans* in two different scenarios: *in vivo*, during a passage through a murine host and *in vitro*, during propagation in liquid culture. Populations passaged *in vivo* showed slower growth and higher rates of chromosome-level genetic variation compared to those grown *in vitro* [122]. These results seem to indicate that a passage through a living host may lead to changes in the *C. albicans* competitive fitness and seems to result in slower growth and higher rates of genomic and phenotypic variation compared to *in vitro* populations. In fact, recently, Tso *et al*. demonstrated that *C. albicans* can be experimentally induced to increase its competitive fitness in the gut of a mouse [125]. In this study, long-term gastrointestinal colonization of antibiotic-treated mice by *C. albicans* was coupled with serial fecal transplants from colonized to naï;ve hosts. After 8-10 weekly serial passages it was verified a significantly increased intra-gastrointestinal competitive fitness, at similar levels to strains deficient in filamentous growth known to have increased fitness in antibiotic-treated mouse gut [126–128]. In fact, all *C. albicans* populations were observed to progressively lose their ability to form true hyphae when evolved in the presence of antibiotics [125].

Another study addressing the adaption of *C. albicans* isolates recovered from cystic fibrosis patients also pointed to morphological changes as a possible host-associated adaption mechanism [129]. In this case, several *C. albicans* isolates were seen to acquire a filamentation phenotype, underlined by mutations in the filamentation transcriptional repressor *NRG1*. Notably, distinct strains acquired dissimilar mutations, albeit all being located before or within the DNA binding domain of *NRG1*. As such, the observed filamentous growth phenotypes were associated to the likely loss of function of Nrg1 [129].

## ADAPTION TO THE HUMAN IMMUNE SYSTEM

### *C. glabrata* evolution towards increased oxidative stress resistance

Upon phagocytosis by the host immune system, pathogens are subject to oxidative burst [130, 131]. *C. glabrata* shows increased tolerance to oxidative stress when compared to other yeasts, including *Saccharomyces cerevisiae* and *C. albicans* [132]. Resistance to oxidative stress and detoxification of Reactive Oxygen Species (ROS) is mainly associated with the activity of the catalase Cta1, the superoxide dismutases Sod1/Sod2 and the glutathione and thioredoxin pathways [132–135]. As shown by several genome-wide studies, regulation of the oxidative stress response is governed by the transcription factor Yap1 *in vitro* [136–139], however, Yap1 does not appear to be a virulence determinant and works in conjunction with other oxidative stress resistance (OSR) determinants to mediate phagocyte survival [137, 140]. The ability of *C. glabrata* to overcome oxidative stress was inspected by *in vitro* evolution during oxidative (H_2_O_2_) challenge [141]. Individual *C. glabrata* populations were serially passaged in the presence of increasing concentrations of H_2_O_2_, resulting in a significant increase of MIC_50_ of H_2_O_2_ in comparison to the initial population. Increased tolerance and resistance to H_2_O_2_ is a result of adaptive evolution to oxidative stress, based on increased survival frequency of the evolved populations. Evolved strains were seen to adapt faster to hydrogen peroxide stress. In addition, adapted strains were capable of faster H_2_O_2_ detoxification and growth in higher concentrations of hydrogen peroxide than the parental strains [141]. The study resorted to whole genome sequencing and transcriptomics analysis of adapted mutants to identify the molecular basis for the evolution of populations to oxidative stress. Whole genome sequencing on selected evolved strains showed a total of 47 mutations (28 inside coding sequences). Interestingly, eleven of the mutated genes are known to affect H_2_O_2_ resistance when inactivated in *S. cerevisiae*. Among them, is a homolog of the *MGA2* transcription factor (*CAGL0F06831g*), a regulator of lipid modulation in *S. cerevisiae* that confers H_2_O_2_ resistance when inactivated in this species [142]. Therefore, the occurrence of a mutation in *C. glabrata MGA2*, taking place in an evolved strain, possibly contributes to changes in membrane fatty acid composition that mediate H_2_O_2_ resistance. In fact, transcriptomics analysis during oxidative stress between evolved strains and their parental strains revealed that modulation of membrane composition is potentially involved in adaptation of *C. glabrata* to H_2_O_2_ [141]. Genes involved in amino acids and fatty acids were commonly upregulated among more than one evolved strain. Additional processes overrepresented in differentially expressed genes comprise cell wall remodeling and NADPH regeneration. Interestingly, only three genes were commonly up-regulated in at least three of the four adapted strains probed in the referred study. All of them have homologs involved in H_2_O_2_ resistance in *S. cerevisiae*. These genes (*YDJ1, PDC1* and *TAL1*) could therefore represent interesting targets to study adaption of *C. glabrata* to oxidative stress. Moreover, a nonsense mutation in *C. glabrata CTH2* was also found in an evolved strain. In both *C. glabrata* and *S. cerevisiae, CTH2* is known for its role in the post-transcriptional regulation of iron limitation response through degradation of mRNAs encoding proteins involved in iron-dependent processes [143, 144]. Nevertheless, increased expression of this gene contributes to enhanced ROS tolerance in *S. cerevisiae* [145]. The mutation of *C. glabrata CTH2* was shown to contribute directly for H_2_O_2_ tolerance, as a reference strain containing the mutated *CHT2* displayed significant more tolerance than the same strain carrying the wild type gene upon exposure to a high concentration of H_2_O_2_ [141].

### *Candida* evolution during macrophage interaction

Interaction with phagocytes is another relevant topic to better understand how pathogens overcome the host immune system. More than just survive the harsh environment of the phagosome, virulence traits include the ability to escape engulfment by phagocytic cells or avoid immune recognition all together.

Despite belonging to the same genus, *C. albicans* and *C. glabrata* display distinct virulence features during macrophage interaction. One of the most striking differences is in their ability or inability to form hyphae. Formation of hyphae by *C. albicans* is associated with several virulence features, namely biofilm formation, host tissue invasion and macrophage escape [10, 12, 14, 15]. On the other hand, *C. glabrata* is regarded to exist mainly in yeast form, forming pseudohyphae under particular conditions and not producing true hyphae [146]. Therefore, it lacks the yeast-to-hyphae dimorphism that comprises one of the main virulence factors of *C. albicans* [147].

Interestingly, a work by Brunke *et al*. has shown that *C. glabrata* cells co-incubated during 6 months with macrophages were able to produce pseudohyphae structures and evolve into a hypervirulent phenotype [148]. Daily passages of *C. glabrata* cells to new macrophage cultures resulted in the appearance of the first altered morphology cells after 1 month. The morphology of the evolved strain consisted of elongated clumps of several cells, resembling pseudohyphae structures. This change was associated with a wrinkled colony phenotype, was found to be genetically stable and to be correlated with changes in the thickness of the cell wall. The macrophage-evolved strain harbored a non-synonymous mutation in the catalytic region of the chitin synthase encoding *CHS2* gene, which was shown to be responsible for its pseudohyphae growth morphology. Moreover, it was associated with a reduction in accessible chitin and reduced content of mannan and β-glucan. During co-incubation with macrophages, the expansion of the evolved strain was seen to increase continuously. As part of macrophage adaptation, the evolved strain presented a fitness advantage in comparison to wild type *C. glabrata* cells; but surprisingly, the increased fitness was not associated with increased tolerance to typical phagosome stress conditions. Another interesting finding was the specific microevolution of *C. glabrata* to host cell types. The evolved *C. glabrata* strain resulting from co-incubation with macrophages was seen to do significant more damage to macrophages than the parental strain after 24h, but the same was not observed for epithelial cells. Higher macrophage damage by the evolved strain was associated with faster escape after phagocytosis. Concordantly, the parental strain took four days to kill macrophages, possibly due to overgrowth of fungal mass; however, the evolved strain was able to burst macrophage cells after only 48h and continue to replicate in pseudohyphal form. These findings indicate that *C. glabrata* is able to evolve in a host cell-type specific manner. The microevolution of *C. glabrata* upon macrophage co-incubation also resulted in increased virulence in comparison to the parental strain, which was dependent on the point mutation in *CHS2* that induced pseudohyphal growth [148].

A similar study by Wartenberg *et al*. has probed the microevolution of a *cph1*∆*/efg1*∆ *C. albicans* strain during macrophage interaction [149]. In this species, *CPH1* and *EFG1* are two of the main regulators of the hyphal regulatory network [150–153]. As a result, the double mutant strain is incapable of hyphal growth and macrophage escape [149]. Surprisingly, microevolution of the referred mutant during co-incubation with macrophages yielded the formation of filamentous structures (mixture of pseudohyphae and true hyphae) after 19 daily passages. This phenotype was also observed in response to several filamentation inducing conditions *in vitro*, including serum or N-acetyl-D-glucosamine as sole carbon source. These findings indicate that microevolution during macrophage coincubation potentiated new pathways for filamentation in *C. albicans*. The regain of filamentation capacity allowed the evolved strain to escape macrophages (from totally incapable in the original mutant), albeit not as efficiently as the wild type [149]. The same trend was verified in terms of adhesion potential and epithelia invasion. An increase in the damage of the evolved strain relative to its original mutant was yet observed for both macrophages and epithelial cells [149]. Cell wall architecture was also partially restored in the evolved strain, reverting the reduced mannan and increased β-glucan levels in the original mutant. It appears to be clear that the absence of two major filamentation regulators is too severe to regain full wild type-comparable virulence, but the fact that the evolved strain was able to recover partial virulence features underscores the importance of microevolution studies in the study of infection by fungal pathogens. Consistent with the previous observations, the evolved strain upregulates hyphal specific genes (e.g., *HWP1, ALS3* and *ECE1*) and the hyphal morphogenesis regulator *UME6* [149]. Moreover, the upregulation of SAP and cell wall biogenesis genes was observed. Altogether, the expression program of the evolved strain appears to underscore the regained ability of filamentous growth and host cell damage. Furthermore, the evolved strain harbored a non-synonymous mutation in the activation segment of the catalytic domain of the kinase *SSN3* gene, which was shown to confer the ability to produce hyphae and damage macrophages after macrophage microevolution, even in the absence of Efg1 and Cph1 [149].

**TABLE 1.** **Reported mechanisms of adaption to antifungal treatment, host niches and immune attack in** ***C. albicans*** **and** ***C. glabrata*** **as determined by microevolution studies**. Both *in vivo* or *in vitro* studies are considered. The main features found to be associated with evolution events in each condition are displayed

Study	Species	Environmental condition	*in vivo*	*in vitro*	Observed changes
Cowen *et al*. 2000 [83]	*C. albicans*	Fluconazole treatment		x	Overexpression of azole resistance genes (*CDR1, CDR2, MDR1, ERG11*)
**Vincent** ***et al***. **2013** [84]	*C. albicans*	Amphotericin B treatment		x	*ERG6* mutation + LOH
**Cavalheiro** ***et al*****. 2019** [85]	*C. glabrata*	Fluconazole treatment		x	*PDR1* GOF + *EPA3* upregulation
**Bordallo-Cardona** ***et al*****. 2018** [88]	*C. glabrata*	Echinocandin treatment		x	*FKS2* mutations
**White** ***et al*****. 1997** [92]	*C. albicans*	Fluconazole treatment		x	ERG11 mutations
**Dunkel** ***et al*****. 2008** [36]**, Heilmann** ***et al*****. 2010** [32]**, Hoot** ***et al*****. 2011** [31],	*C. albicans*	Fluconazole treatment	x		UPC2 GOF, leading to ERG11 upregulation
**Coste** ***et al*****. 2006 [35]**	*C. albicans*	Fluconazole treatment		x	TAC1 GOF + LOH
**Coste** ***et al*****. 2007** [94]	*C. albicans*	Fluconazole treatment	x		TAC1 and ERG11 mutations + LOH
**Popp** ***et al*****. 2017** [95]	*C. albicans*	Fluconazole treatment	x		*TAC1* GOF, *MRR1* GOF leading to *MDR1* upregulation
**Dunkel** ***et al*****. 2008** [34]	*C. albicans*	Fluconazole treatment	x	x	*MRR1* GOF + LOH, leading to *MDR1* upregulation
**Morschhäuser** ***et al*****. 2007** [99]	*C. albicans*	Fluconazole treatment	x		MRR1 *GOF, leading to* MDR1 upregulation
**Ford** ***et al*****. 2015** [100]	*C. albicans*	Fluconazole treatment	x		Mutations (several) + LOH
**Vale-Silva** ***et al*****. 2017** [86]	*C. glabrata*	Fluconazole treatment	x		PDR1 GOF
**Singh-Babak** ***et al*****. 2012** [103]	*C. glabrata*	Echinocandin treatment	x		FKS2 mutation
**Biswas** ***et al*****. 2017** [106]	*C. glabrata*	Multidrug treatment	x		*FKS1* and *FKS2* mutations (echinocandin), *PDR1* GOF (fluconazole)
**Lüttich** ***et al*****. 2013** [124]	*C. albicans*	Murine kidney	x		Resistance to oxidative stress and high temperature
**Cheng** ***et al*****. 2007** [121]	*C. albicans*	Murine spleen	x		Uncoupling of oxidative phosphorylation + resist phagocyte killing
**Forche** ***et al*****. 2009** [122]	*C. albicans*	Murine kidney	x		Chromosome rearrangements + altered colony morphology
**Tso** ***et al*****. 2018** [125]	*C. albicans*	Murine gut (antibiotic-treated mice)	x		Increased competitive fitness + loss of hyphae formation
**Kim et al. 2015** [129]	*C. albicans*	Sputum (cystic fibrosis patients)	x		NRG1 mutation
**Huang and Kao** ***et al*****. 2018** [141]	*C. glabrata*	OSR	x		CTH2 mutation
**Brunke** ***et al*****. 2014** [148]	*C. glabrata*	Macrophage escape		x	*CHS2* mutation
**Wartenberg** ***et al*****. 2014** [149]	*C. albicans*	Macrophage escape		x	*SSN3* mutation

## CONCLUSIONS

The use of microevolution experiments has proven to be a relevant research topic to expand knowledge on how to tackle infections by *Candida* species. In this review, the data achieved by microevolution experiments to antifungal treatment, biofilm formation and host-associated stresses has been detailed.

Resistance to azoles in *C. glabrata* is largely credited to the occurrence of GOF mutations and resulting hyperactivity of the transcription factor Pdr1. Similarly, the easily acquired mutations in *FKS* genes are the most described mechanism of echinocandin acquired resistance in *C. glabrata*. In *C. albicans*, resistance to azoles develops in a stepwise manner with acquired mutations in drug resistance genes, such as *TAC1, MRR1* and *ERG11*, followed by LOH events and additional gain of identical copies of the mutated genes. So far as we know, studies regarding *C. albicans* resistance evolution toward echinocandins are yet to be performed.

The information relative to evolution of biofilm formation is much more limited, but current data seems to show that the ability to form biofilm could be intrinsic to a given isolate, once the amount of biofilm formation does not appear to change in most cases over the course of an infection in each patient. Studies addressing the molecular basis that supports differential biofilm production across distinct isolates could help to pinpoint key mechanisms for biofilm formation and potentially the most relevant therapeutic targets. Moreover, co-infection with other species seems to influence this trait.

Evolution of *C. albicans* in the human host appears to result in genotypic and phenotypic variability, possibly as an approach to select for advantageous features. It was reported that *C. albicans* suffers changes in its competitive fitness, including changes in energy metabolism, reduction of growth rates and attenuated virulence, possibly as a persistence strategy. When probing evolution during immune encounter, both *C. albicans* and *C. glabrata* escape macrophage phagocytosis by producing hyphae or pseudohyphae, respectively. In both species, the production of pseudohyphae was associated with changes in cell wall and consistent with morphological changes. Moreover, the evolution to produce filaments was associated with mutations in key genes encoding: the chitin synthase Csh2 in *C. glabrata* and the Ssn3 kinase in *C. albicans*.

Interestingly, both *C. albicans* and *C. glabrata* appear to rely heavily on the occurrence of mutations, ultimately selecting a SNP or a set of SNPs that confer an evolutionary advantage. Also, this strategy takes place in more than just one selective pressure, indicating that is one of the preferred mechanisms for microevolution of these pathogens. The data from experimental evolution emphasizes the notion that resistance is a multifactorial process, often achieved in a stepwise fashion and through the combination of multiple mechanisms (**Table1**; **Figures 1** and **2**). Additionally, it is also possible that serial isolates from the same patient can result in resistance caused by different trajectories, which emphasizes the need to better understand evolutionary dynamics.

More than just identifying possible mechanisms of adaptation to most stress conditions human pathogens encounter, it is important to clinically validate the experimentally obtained clues. One good example of the occasional inconsistency between lab and clinical data is the participation of *C. glabrata ERG11* gene in azole resistance. Although this was observed in laboratorial experiments, through *ERG11* upregulation [154, 155] or increased protein abundance [156], however, these mechanisms do not appear to play such a prominent role in clinical resistant isolates [41, 157, 158]. On the other hand, additional mechanisms of echinocandin resistance other than *FKS* mutations are starting to be identified [159], but the assessment of such cases in large isolate collections needs to be conducted in order to understand their clinical significance in comparison to *FKS* mutations. The full understanding of stress adaptation in human pathogens requires the integration of several sources of evidence to fully understand the most relevant resistance mechanisms, their regulation, their activation conditions and how they evolve in the clinical setting.

## Abbreviations:

AmB: amphotericin B,
DMCDD: 14,24-dimethylcholesta-8,24(28)-dien-3β,6α-diol,
EMC: extracellular matrix,
EPS: extracellular polymeric substances,
GFO: gain of function,
LOH: loss of heterozygosity,
NGS: next-generation sequencing,
ROS: reactive oxygen species,
SAP: secreted aspartyl proteases,
SNP: single nucleotide polymorphism,
VERT: visualizing evolution in realtime.
